# mTOR inhibition improves mitochondria function/biogenesis and delays cardiovascular aging in kidney transplant recipients with chronic graft dysfunction

**DOI:** 10.18632/aging.202863

**Published:** 2021-03-23

**Authors:** Barbara Infante, Francesco Bellanti, Michele Correale, Paola Pontrelli, Rossana Franzin, Serena Leo, Martina Calvaruso, Silvia Mercuri, Giuseppe Stefano Netti, Elena Ranieri, Natale Daniele Brunetti, Giuseppe Grandaliano, Loreto Gesualdo, Gaetano Serviddio, Giuseppe Castellano, Giovanni Stallone

**Affiliations:** 1 Department of Medical and Surgical Sciences, Nephrology, Dialysis and Transplantation Unit, University of Foggia, Foggia, Italy; 2 C.U.R.E. (University Center for Liver Disease Research and Treatment), Department of Medical and Surgical Sciences, University of Foggia, Italy; 3 Cardiology Unit, Department of Medical and Surgical Sciences, University of Foggia, Foggia, Italy; 4 Nephrology, Dialysis and Transplantation Unit, Department of Emergency and Organ Transplantation, University of Bari “Aldo Moro”, Bari, Italy; 5 Clinical Pathology Unit and Center for Molecular Medicine, Department of Medical and Surgical Sciences, University of Foggia, Foggia, Italy; 6 Fondazione Policlinico Universitario A. Gemelli IRCCS, Rome, Italy; 7 Università Cattolica del Sacro Cuore, Rome, Italy

**Keywords:** cardiovascular aging, mTOR inhibitor, kidney transplantation, mitochondria, kidney disease

## Abstract

CVD remains the major cause of mortality with graft functioning in Kidney transplant recipients (KTRs), with an estimated risk of CV events about 50-fold higher than in the general population. Many strategies have been considered to reduce the CV risk such as the use of mTOR inhibitors. We evaluate whether chronic mTOR inhibition might influence CV aging in KTRs studying the molecular mechanisms involved in this effect. We retrospectively analyzed 210 KTRs with stable graft function on therapy with CNI and mycophenolic acid (Group A, 105 pts.), or with CNI and mTORi (Everolimus, Group B, 105 pts.). The presence of mTOR inhibitor in immunosuppressive therapy was associated to increase serum levels of Klotho with concomitant reduction in FGF-23, with a significant decrease in left ventricular mass. In addition, KTRs with mTORi improved mitochondrial function/biogenesis in PBMC with more efficient oxidative phosphorylation, antioxidant capacity and glutathione peroxidase activity. Finally, group B KTRs presented reduced levels of inflammaging markers such as reduced serum pentraxin-3 and p21ink expression in PBMC. In conclusion, we demonstrated that mTOR inhibition in immunosuppressive protocols prevents the occurrence and signs of CV aging in KTRs.

## INTRODUCTION

Patients with chronic kidney disease (CKD) face a dramatically increased risk of mortality from cardiovascular disease (CVD) [1, 2]. Structural and functional alterations of the cardiovascular (CV) system characterize CKD including endothelial dysfunction, arterial stiffening and changes in left ventricular geometry, closely resembling the modifications of vessels wall and heart of aging subjects [3]. Indeed, CKD can be considered as a model of accelerated CV aging [2, 4–6], since heart and vessels are more susceptible to several stress conditions [7]; with development several hemodynamic changes, hypertension and atherosclerosis. Interestingly, this process appears to be independent by traditional cardiovascular risk factors, but linked to accelerated aging phenomena [8].

Mitochondria may represent a further link in this contest, since mitochondrial dysfunction has been reported in different models of CKD [9]. These subcellular organelles play a key role in the aging process modulating cellular oxidative stress, although there is now evidence of alternative pathways of aging-associated mitochondrial dysfunction leading to functional decline of cells independent of reactive oxygen species [9]. Klotho, a fibroblast growth factor- 23 (FGF-23) co-receptor, has a well-recognized function in the aging process and may represent a link between aging and kidney disease [10]; Klotho plays a key role in the regulation of aging, particularly at the cardiovascular level, by inhibiting local phosphate uptake into vascular smooth muscle cells (VSMCs), and their differentiation in osteoblast-like cells and preservation of endothelial function [11, 12].

Mammalian target of rapamycin (mTOR) is a key cellular kinase, whose inhibition has been associated with an increased lifespan in worms, flies and mice [13, 14]. In addition, mTOR modulates several metabolic pathways contributing to cellular senescence, including autophagy, mitochondrial respiratory function and biogenesis, and Klotho expression/function [15, 16]. Moreover, mTOR is up-regulated by nutrients, including phosphate, that induces vascular calcification, driving osteogenic trans-differentiation of VSMCs [17]; interestingly, mTOR activation in VSMCs can reduce expression of klotho [17, 18].

Specifically, in the setting of kidney transplant recipients (KTRs), CVD remains the major cause of mortality with functioning graft [19], with an estimated risk of CV events about 50-fold higher than in the general population [20]. I Immunosuppressive therapy can adversely affect kidney function; moreover, immunosuppressive drugs might lead to relevant side effects linked to CVD with the development of unfavorable CV scenario in KTRs [21, 22]. Many strategies have been considered to reduce the CV risk of KTRs, such as steroids or calcineurin inhibitors (CNI) minimization, but current data are inconclusive [23]. The cornerstone of CNI minimization is the introduction of mTOR inhibitors (mTORi) in the immunosuppressive protocol [24–26].

In this study we investigated whether chronic mTOR inhibition might influence CV aging in KTRs; moreover, we evaluated the molecular mechanisms involved in this effect, focusing on the role of klotho/FGF23 axis and mitochondrial function/biogenesis.

## MATERIALS AND METHODS

### Patients

We conducted a multicenter, observational, case–control study enrolling KTRs in Transplantation centers of Foggia and Bari (Italy). The study protocol was approved by the Ethical Committee of the coordinating center (Study n° 4440; Prot. N.670/CE−2014) according to the Declaration of Helsinki. Once written informed consent was collected, we included in the study 210 renal transplant recipients with stable graft function and affected by CKD stage 3–5, on therapy with CNI and mycophenolic acid (Group A, 105 pts.), or with CNI and mTORi (Everolimus) (Group B, 105 pts.) from at least one year. The patients were recruited after qualifying to the following criteria: inclusion criteria: age>18 years; recipient of a primary renal allograft from a cadaveric donor, stable graft function, calcineurin-inhibitors-based therapy; estimated GFR<60 ml/min, time from transplantation >12 and <60 months; leukocytes>4x109/L, platelets>100x109/L, fasting triglycerides<350mg/dL, fasting cholesterol<300mg/dL. Exclusion criteria: panel reactive antibodies >50%; an acute rejection episode in the previous 12 months; active systemic infection [27]; presence of neoplasia and previous history of cancer [28].

No changes in immunosuppressive therapy were done from at least one-year former enrollment for all patients recruited. The enrollment period was 12 months and follow-up time was two years monitoring the principal CV events. The immunosuppressive efficacy was monitored through the measurement of the drugs trough level (T.L.). The two groups were matched for the main demographic features, graft function and transplantation vintage.

### Methods

### *Cardiac evaluation*

All patients were evaluated by conventional 2D and TDI echocardiography under resting conditions as previously described [29].

Transthoracic echocardiography was performed with the use of iE33 (Philips Medical Systems, Andover, MA, USA) as previously described [30, 31].

TDI was performed using apical views for the long-axis motion of the ventricles. Two-dimension echocardiography with TDI-color imaging was performed using aS5-1 Sector Array Transducer with Pure Wave Crystal Technology (5 to 1 MHz). Two-dimensional echocardiography with TDI color imaging views were optimized for pulse repetition frequency, color saturation, sector size, and depth and were allowed the highest possible frame rate. At least 3 consecutive beats were stored, and the images were analyzed offline with the aid of a customized software package (QLAB quantification software, Philips).

### PBMC isolation and RNA extraction

Twenty milliliters of whole blood were collected from patients (8 CNI-MPA and 10 CNI-EVE) at the time of enrollment (T0). For 5 out of 8 CNI-MPA patients, whole blood was harvested at T0 and after 6 (T1) and 12 months (T2) from conversion to Everolimus treatment. PBMCs were isolated by density separation over a Ficoll–Hypaque gradient (Flow-Laboratories, Irvine, UK). Total RNA was extracted by the RNeasy mini kit (Qiagen, Valencia, CA).

### Real-time quantitative qPCR

Reverse transcription of total RNA (500 ng) was performed as previously described [32]. qPCR was conducted in triplicate using SsoAdvanced™ Universal SYBR® Green Supermix (Bio-Rad Laboratories, Hercules, CA, USA) and two separate sets of oligonucleotide primers specific for Homo sapiens cyclin-dependent kinase inhibitor 1A (CDKN1A), transcript variant 1, mRNA (p21), (upstream 5’- TGGAGACTCTCAGGGTCGAAA-3’, downstream:5′ GGCGTTTGGAGTGGTAGAAATC-3’ Invitrogen, Milan, Italy) and glyceraldehyde-3 phosphate dehydrogenase (GAPDH) (upstream 5′-GAA GGT GAA GGT CGG AGT CA-3′; downstream: 5′-CAT GGG TGG AAT CAT ATT GGA A-3′; Invitrogen, Milan, Italy) [33] (Light Cycler@96 instrument (Roche, Mannheim, Germany) was programmed with an initial step of 30 seconds at 95° C, followed by 40 thermal cycles of 15 seconds at 95° C and 60 seconds at 60° C for GAPDH and p21. Melting curve analysis was employed to exclude nonspecific amplification products. The comparative *C*_t_ method (ΔΔ*C*_t_) was used to quantify gene expression and the relative quantification (RQ) was calculated as 2^−ΔΔ^*^C^*^t^.

### Klotho/FGF23 and PTX-3 serum level assessment

Circulating Klotho/FGF23 and PTX3 were measured by ELISA, according to the manufacturer's instructions (R&D Systems, Minneapolis, MN).

### Mitochondrial function/biogenesis

### *Laboratory measurements*

Blood samples were obtained and treated as previously described [34].

Serum fluorescent adducts formed between peroxidation-derived aldehydes (HNE and MDA) and proteins were measured by spectrofluorimetry as previously reported [35]. The antioxidant activity was measured as Trolox equivalent antioxidant capacity (TEAC) in blood, according to a previously published method [36]. Glutathione peroxidase (GPx) activity was analyzed in PBMCs by spectrophotometry following the Cayman Assay kit procedure (n° 703102).

### *Measurement of respiratory activity*

We resuspended freshly isolated PBMCs at 1 × 10^6^cells/500 μl in 10 mM KH_2_PO_4_, 27 mM KCl, 1 mM MgCl_2_, 40 mM HEPES, 0.5 mM EGTA (pH 7.1) and evaluated O_2_ consumption by high resolution respirometry (Clark electrode, Hansatech Instruments Ltd, Norfolk, UK) at 37° C under continuous stirring. Next, we added oligomycin (8 μg/ml), followed by 5 min by the addition of valinomycin (2 μg/ml). Oxygen consumption rate (OCR) was corrected for 3 mM KCN-insensitive respiration and normalized to the cell number. finally, we calculated respiratory control ratio (RCR) by dividing the rates of oxygen consumption obtained before and after the addition of oligomycin.

### *Flow cytometric analysis*

We investigated mitochondrial injury and alteration in membrane potential (Δψ) by staining with 5,5′,6,6′-tetrachloro-1,1′,3,3′-tetrathylbenzimidazolyl carbocyanine iodide (JC-10; Abcam Inc, Cambridge, UK),.

We detected reactive species by using 2′,7′-dichlorofluorescein diacetate (DCFH-DA, Sigma-Aldrich, St Louis, Missouri, USA). We washed the cells with PBS and incubated with 10 μM DCFH-DA for 30 min at 37° C. Analysis was carried out by a Flow Sight Imaging flow cytometer (Luminex Corporation, MV ‘s-Hertogenbosch, the Netherlands).

### Statistical analysis

Data were expressed as mean ± standard deviation of the mean (SDM). Gaussian distribution of the samples was evaluated by Kolmogorov–Smirnov test. The significance of differences between the two groups was assessed by the student’s t-test for unpaired samples (SPSS, Inc., Chicago, IL; GraphPad Software, Inc., San Diego, CA).

We expressed continuous variables as mean ± standard deviation, and categorical variables as percentages. We compared mean values with Student's t-test for variables with normal distribution or with the Mann–Whitney non-parametric U test for variables with a non-normal distribution. Percentages were compared with χ2 test. A p<0.05 was considered statistically significant.

## RESULTS

### Demographic and biochemistry data

We did not observe statistical differences between the two groups of patients at the end of follow-up regarding age, transplant age, graft function, acute rejection episodes, appearance of de Novo Donor Specific antibodies, blood levels of immunosuppressive drugs, as well as for leukocytes, platelets, triglycerides, cholesterol, parathormone (PTH) and 1,25 dihydroxycholecalciferol (Table 1).

**Table 1:** Demographic, clinical and biochemistry data.

	**T0**	**Group A**	**Group B**	**T24**	**Group A**	**Group B**	**P value**
Number		105	105		105	105	NS
HLA Mismatches (n°)		3 + 1	2.8 + 1.3		3 + 1	2.8 + 1.3	NS
GFR<60 ml/min		42 + 14	39 + 19		40 + 9	41 + 16	NS
Time from transplantation (mo)		26.57 + 5.04	29.17 + 5.9		50.57 + 5.08	53.17 + 5.88	NS
Age (yrs)		48.18 + 8.18	53.75 + 6.09		50.18 + 8.2	55.75 + 6.1	NS
Acute rejection episodes (n°)		0	0		0	0	NS
Fasting triglycerides (mg/dl)		198 + 39	221 + 68		198 + 39	221 + 88	NS
Fasting cholesterol (mg/dl)		175 + 48	196 + 39		188 + 32	202 + 27	NS
White blood cells (mmc)		8752 + 1263	6523 + 985		7120 + 1452	6900 + 1159	NS
Platelets (mmc)		291 + 111	269 + 124		275 + 97	212 + 74	NS
PTH (pg/ml)		289 + 99	304 + 87		312 + 114	348 + 107	NS
1,25 VIT D (ng/ml)		23.4 + 11.3	28.2 + 13.7		20.4 + 12.2	22.6 + 15.7	NS
De novo DSA %		0	0		0	0	NS
Immunosuppressive Therapy		TAC – MMF - CS	TAC - EVR - CS		TAC – MMF - CS	TAC - EVR - CS	NS
T.L. Tacrolimus (ng/ml) T.L. Everolimus (ng/ml)		6.8 + 2.1	4.8 + 1.6		6.1 + 0,9	4.2 + 1.9	NS
		0	3.8 + 1.1		0	3.1 + 0,7	NS

**Figure 1:**
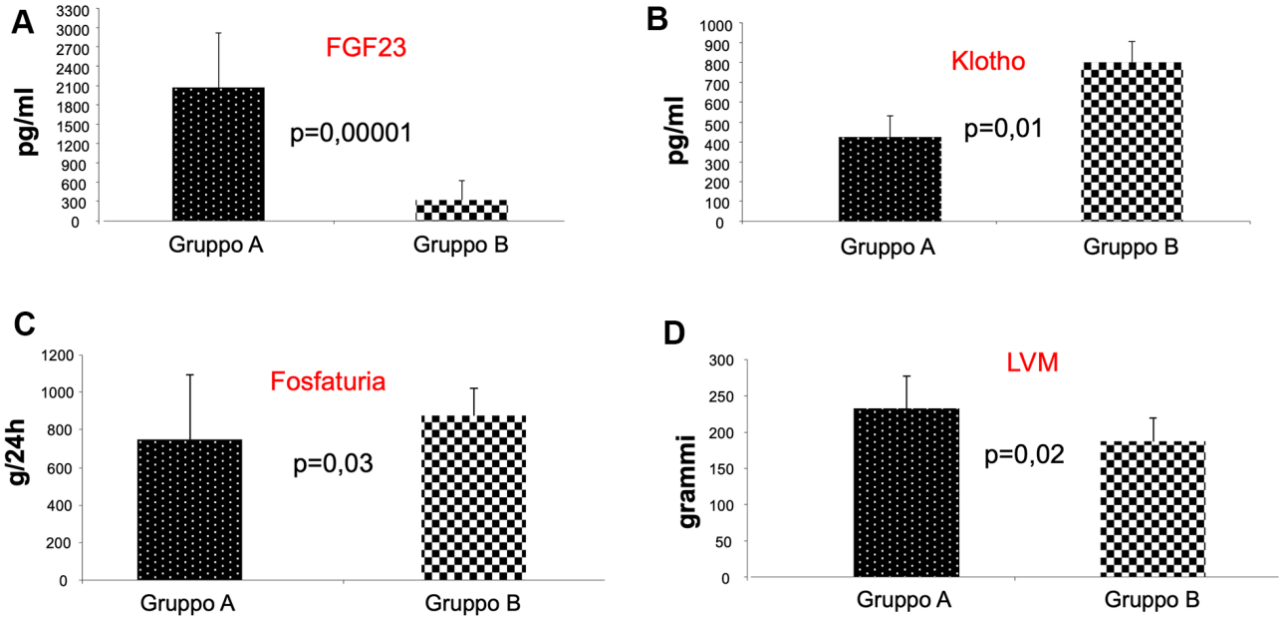
**Analysis of FGF-23, klotho, and phosphate urine excretion in the two groups of patients.** As shown, we observed a significant statistical difference between the two groups of patients for the values of FGF-23, Klotho, phosphate urine excretion, indicating that patients receiving mTORi Group (**B**) presented reduced FGF-23 levels (**A**), increased Klotho levels (**B**) with enhanced phosphaturia (**C**). Interestingly, we also found a reduced left ventricular mass (LVM) in group B patients (**D**).

Interestingly, we observed a significant statistical difference between the two groups of patients for the values of FGF-23, Klotho, phosphate urine excretion (FGF-23 pg/ml; 2068,2 + 846,5; 320,7 + 191; P=0.00001; Klotho pg/ml 425,8 + 102; 802 + 109; p=0.01; phosphate urine excretion uPg/24 751,091 + 342,659; 878,142 + 270,913; p=0.03, A vs B) (Figure 1A–1C, respectively). The presence of mTOR inhibitor in immunosuppressive therapy was associated with an increase Klotho levels with a concomitant reduction in FGF-23 levels; we did not find differences in PTH and 1,25 dihydroxycholecalciferol, demonstrating that klotho-FGF-23 axis in the kidney supports the phosphate homeostasis inducing phosphate urine excretion.

### Cardiac evaluation

When we analyzed cardiac parameters, we did not observe any difference in the two groups regarding pulse wave velocity (PWV), Flow Mediated Dilation (FMD), ejection fraction (EF), LV-filling velocity and E-deceleration time (EDT), the ratio of trans-mitral early to late (E/A ratio), peak velocities of trans-mitral early (E) and late diastolic (A) LV filling. Moreover, none of patients had experiences of serious CV events before and during the follow-up period.

In contrast, we observed a significant statistical difference between the two groups of patients in left ventricular mass (LVM) (gr; 232,7 + 45; 187,8 + 31; p=0.02, A vs B) (Figure 1D). The cardiac evaluation showed that only the value of LVM was reduced in patients of group B compared with Group A. This result underlines the protective effect of mTOR inhibitors on heart function, because LVM is the strongest predictor of the risk of subsequent congestive heart failure, a CV complication that can negatively affects the outcome of KTRs.

### Mitochondrial function/biogenesis

**Figure 2:**
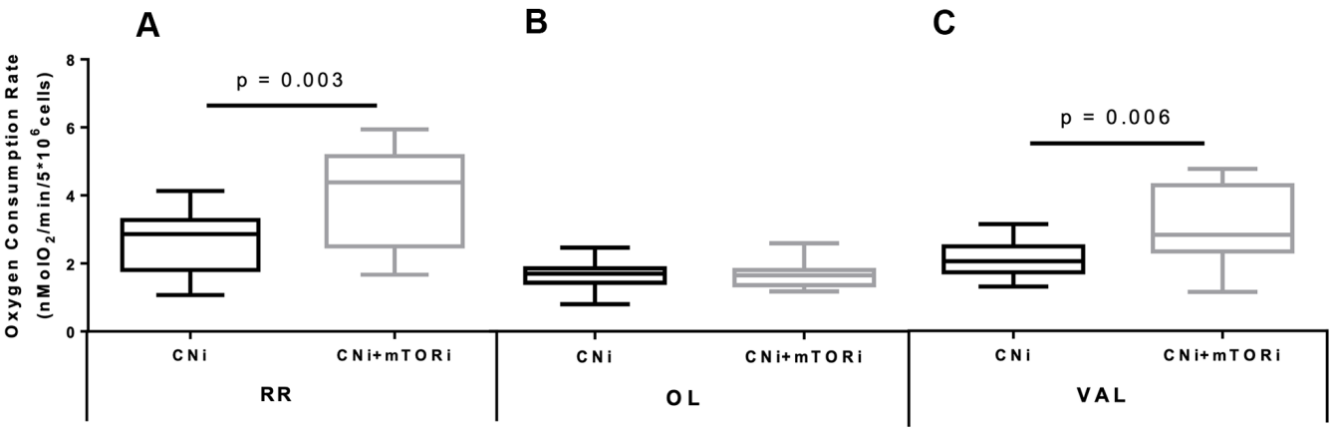
Boxplot representation of the normalized and KCN-insensitive-corrected oxygen consumption rates measured in peripheral blood mononuclear cells (PBMC) from patients treated with calcineurin inhibitor (CNi) or CNi + mTOR inhibitor (CNi+mTORi) under resting conditions (RR, **A**), in the presence of oligomycin (OL, **B**) and in the presence of valinomycin (VAL, **C**). Statistical difference was assessed by unpaired student’s t-test.

Next, we analyzed the oxygen uptake of isolated PBMCs by high resolution respirometry. We found a significant increased oxygen consumption rate (OCR) in resting conditions (RR) in the CNI+mTORi group compared with CNI-MPA (Figure 2A). The addition of the F_O_F_1_-ATP synthase inhibitor oligomycin (OL) led to a significant OCR depression in both groups, suggesting that a significant part of mitochondrial respiration was coupled to ATP synthesis (Figure 2B). The collapse of the mitochondrial transmembrane potential (ΔΨm) by the addition of the K^+^-ionophore valinomycin (VAL) restored the OCR to the levels of resting respiration in both groups (Figure 2C). We found that these activities were strongly inhibited by KCN and therefore linked to the mitochondrial respiratory chain.

**Figure 3:**
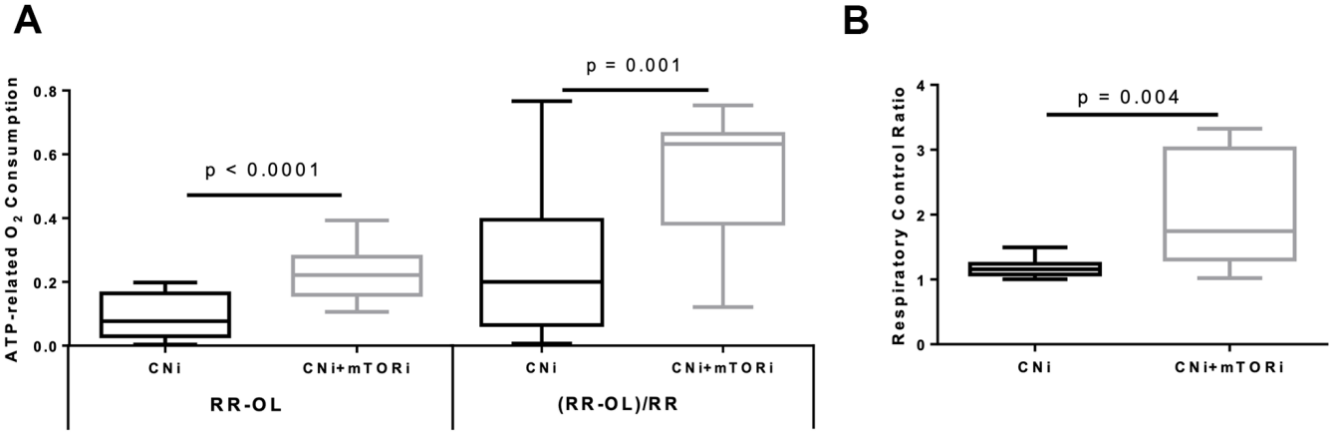
(**A**) Boxplot representation of the ATP-dependent O2 consumption, measured in peripheral blood mononuclear cells (PBMC) from patients treated with calcineurin inhibitor (CNi) or CNi + mTOR inhibitor (CNi+mTORi) as absolute difference between that obtained in the absence and that in the presence of oligomycin (RR-OL) or normalized to basal respiration ((RR-OL)/RR). (**B**) Boxplot representation of the respiratory control ratio obtained by dividing the oxygen consumption rates measured in PBMCs from patients treated with CNi or CNi+mTORi under resting conditions by that in the presence of oligomycin (RR/OL). Statistical difference was assessed by unpaired student’s t-test.

The difference between the RR and after OL addition represents the ATP-related oxygen consumption. As shown in Figure 3A, this rate was increased in PBMCs from patients treated with CNI+mTORi compared with the CNI-MPA group. Similarly, the respiratory control ratio (RCR), which is the ratio between the resting respiration and oxygen uptake after OL addition, was higher in the CNI+mTORi group rather than CNI, indicating more efficient oxidative phosphorylation (Figure 3B).

**Figure 4:**
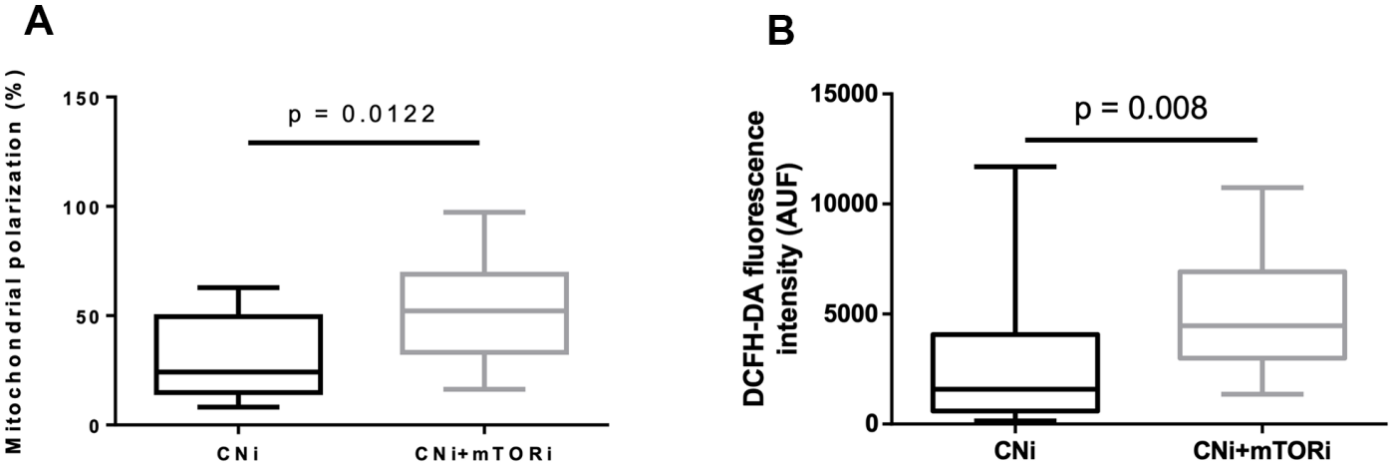
(**A**) Percentage of peripheral blood mononuclear cells (PBMC) with polarized mitochondria from patients treated with calcineurin inhibitor (CNi) or CNi + mTOR inhibitor (CNi+mTORi). Mitochondrial polarization was detected by flow cytometry analysis of cells stained with JC-10. (**B**) Cytofluorimetric detection of reactive species in PBMCs from patients treated with CNi or CNi+mTORi, after staining with 2′,7′-dichlorofluorescein diacetate (DCFH-DA); AUF, arbitrary units of fluorescence. Statistical difference was assessed by unpaired student’s t-test.

The cytofluorimetric analysis of mitochondrial membrane potential highlighted significant differences between the PBMC groups, reflecting a significant higher mitochondrial polarization in CNI+mTORi treated patients than CNI-MPA (Figure 4A). To determine the intracellular production of reactive species, DCFH-DA staining was performed on PBMCs from the same groups. Flow cytometry analysis showed a significantly higher level of fluorescent cells in patients treated with CNI+mTORi rather than CNI-MPA (Figure 4B).

**Figure 5:**
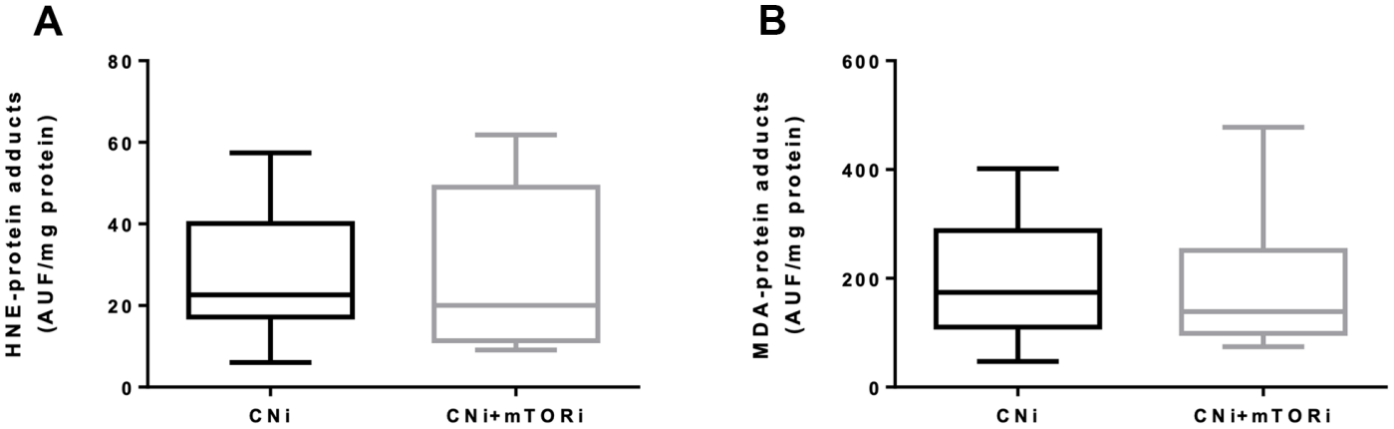
Serum levels of fluorescent hydroxynonenal- (HNE, **A**) and malondialdehyde- (MDA, **B**) protein adducts in patients treated with calcineurin inhibitor (CNi) or CNi + mTOR inhibitor (CNi+mTORi). AUF, arbitrary units of fluorescence.

To verify whether the increased production of reactive species in PBMCs from patients treated with CNI+mTORi was related to circulating oxidative stress, the level of protein oxidation was measured in terms of serum HNE- and MDA-protein adducts (Figure 5). We did not observe any difference in the level of aldehyde-protein adducts between the two groups, suggesting that, despite a higher production of reactive species by the PBMC from patients treated with CNI+mTORi, no circulating oxidative damage was observed.

**Figure 6:**
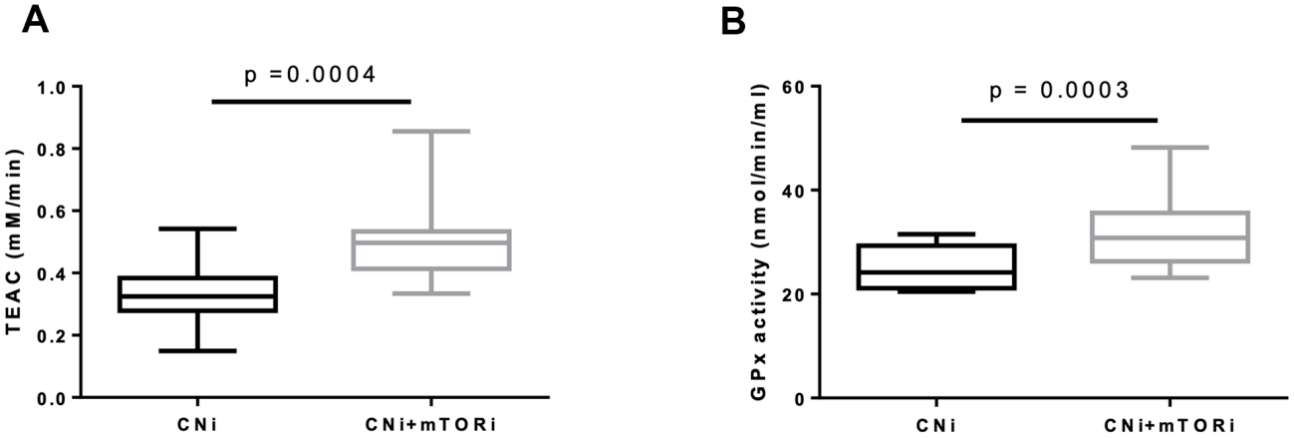
(**A**) Trolox equivalent antioxidant capacity (TEAC) levels in blood from patients treated with calcineurin inhibitor (CNi) or CNi + mTOR inhibitor (CNi+mTORi). (**B**) Glutathione peroxidase (GPx) activity in peripheral blood mononuclear cells (PBMC) from patients treated with CNi or CNi+mTORi. Statistical difference was assessed by unpaired student’s t-test.

Thus, the systemic antioxidant defense in terms of blood trolox equivalent antioxidant capacity (TEAC) and glutathione peroxidase (GPx) activity in PBMC were studied. Interestingly, both TEAC and GPx activity were increased in the CNI+mTORi group with respect to CNI (Figure 6A, 6B).

### Inflammaging and senescence markers

**Figure 7:**
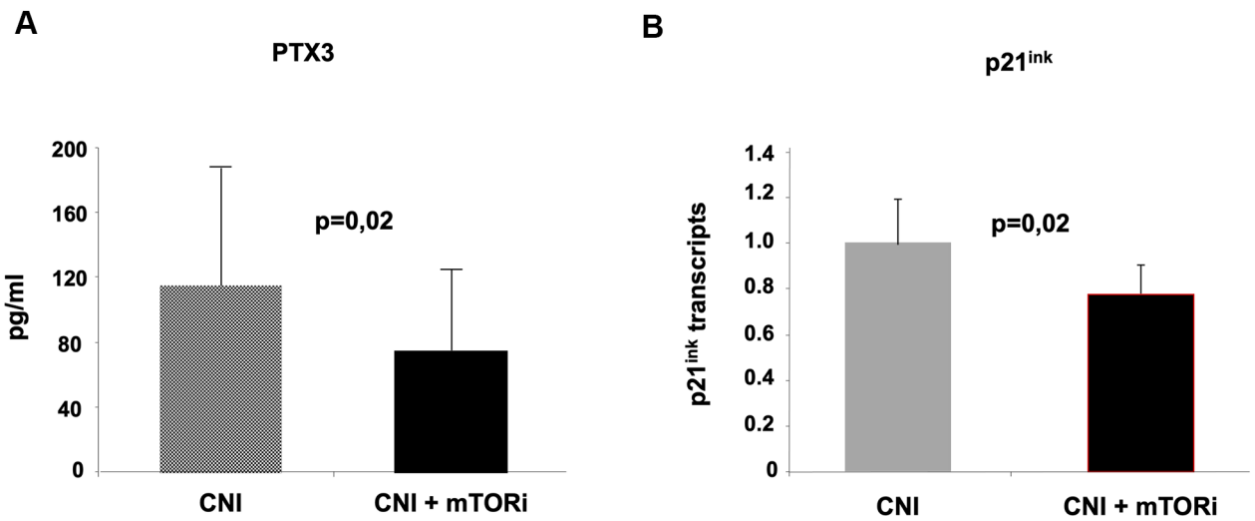
**Evaluation of pentraxin-3 and p21^ink^ as markers of inflammaging in the two groups of kidney transplant recipients.** The inhibition of mTOR pathways in group (**B**) was associated with reduced circulating levels of Pentraxin-3 (**A**) and down-regulation of p21 in PBMC (real-time PCR).

We finally evaluated the effect of mTOR inhibitors on two of the principal markers of Inflammaging and cellular Senescence, Pentraxin-3 and p21^ink^. Interestingly, we observed a statistically significant difference between the values of these markers in the two groups of patients (PTX3 pg/ml 115,73+48,14 vs 76,2+34,21; p=0.02; p21^ink^ pg/ml 1,03+0,12 vs 0.80+0,15; p=0.002 A vs B) (Figure 7A, 7B, respectively). These results suggested that the inhibition of the mTOR pathway could block leucocyte transformation into pro-inflammatory and senescence phenotype. Real-time PCR also confirmed the down-regulation of p21 in PBMCs from group B patients compared to group A (Figure 7B).

## DISCUSSION

In this study, we demonstrated that mTOR inhibition prevents the occurrence and signs of CV aging in kidney transplant recipients. Our results acquired a particular significance because they have been obtained in a high-risk patient population, such as kidney transplant patients affected by chronic graft dysfunction, a population similar to CKD patients.

Several lines of evidence demonstrated that impaired renal function led to increased CV risk in KTRs [37–41]. A post hoc analysis of the FAVORIT Trial showed an association of lower eGFR (less than 45 ml/min/1.73 m^2^) with CV adverse events [42]; in a post hoc analysis of the ALERT study, decreased renal function was associated with increased risk of cardiac death [43]. These findings are further supported by the PORT study in KTRs showing a correlation between lower eGFR and increased coronary heart disease [44]. In this context, immunosuppressive therapy holds a particular importance. It is well known that CNI therapy is closely associated with worsening of renal function. Chronic CNIs nephrotoxicity, which is largely nonspecific in appearance, progresses over time [45, 46]. Although evidence showed less nephrotoxic effect of tacrolimus compared to cyclosporine in this setting of patients [47], however, either CNIs seem to show similar toxicity profile in terms of progressive eGFR reduction [48]. These facts strengthen our study model since all our patients were affected by CKD and with CNIs based therapy with a unique difference being the presence/absence of mTORi. Then, our results demonstrate that the presence of mTORi in the immunosuppressive protocol improves heart function, through the reduction of LVM, the strongest predictor of congestive heart failure that negatively affects the outcome of KTRs.

Another important issue of our results is the effect of mTORi on Klotho/FGF23 axis. Klotho is probably the main ‘aging suppressor’ gene; its defect leads to multiple aging-like phenotypes with premature death around 2 months of age [13, 49]. Klotho is a trans-membrane protein mainly express in the kidney where it works as a co-receptor for FGF-23 [50] mediating several functions [51]. In KTRs, mTORi influences phosphate homeostasis and prolongs hypophosphatemia that usually occurs in the early post-transplant period [52]. It has been demonstrated the mTOR activation can up regulate phosphate transport across the apical membrane of proximal tubular epithelial cells by the Na+-coupled phosphate transporter; interestingly inhibition of mTOR can abrogates this effect [53]. The fact that we did not note a difference in phosphate serum levels between the two groups of patients may be due to an adaptive mechanism typically of CKD. Moreover, our data identify a direct effect of the mTORi on increased urine phosphate excretion, in spite of a reduction in FGF-23 serum level, as suggested by Tataranni et al. [54].

Several evidences suggest a direct and significant correlation between CVD and Klotho expression. Klotho can inhibit local phosphate uptake into VSMCs, counteract matrix mineralization and suppress osteoblast-like differentiation of VSMCs; moreover, Klotho can effici4ntly preserve endothelial function [11, 12, 49]. interestingly, mTOR-inhibitors can be considered as up-regulators of klotho, potentially preventing or delaying the onset of age-related cardiovascular dysfunctions, as suggested by our results.

The most relevant issue of our work was the results obtained from the study of mitochondrial function/biogenesis. We showed a significantly increased oxygen consumption rate in patients treated with mTORi, as well as the respiratory control ratio (RCR), indicating a more efficient oxidative phosphorylation in this group. Moreover, the increase in intracellular production of reactive species, observed in the mTORi treated group, was not correlated with circulating oxidative damage. Interestingly, the systemic antioxidant defense was increased in the mTORi group. Finally, our findings reflected a significantly higher mitochondrial polarization that is an important sign of cellular efficiency. This is an important viewpoint since cardiomyocytes must rely on a constant supply of high-energy phosphates from mitochondria to maintain physiological contractile function of the heart; as consequence, the dysregulation of their homeostasis may play a pivotal role in the onset and development of aging-related cardiovascular disorders [55]. Mitochondria are indeed the primary source of ROS, which triggers endogenous process of apoptosis;; interestingly, this is considered the leading cause of cell death in aging cardiomyocytes [56–59]. In addition, in presence of increased ROS production, the Nrf-2 activity might be inhibited with a subsequent increase in TNF-*α* level:; this will lead to upregulation in Nox complex expression and activity, generating a vicious cycle [58]. Interestingly, there are organs like the heart that are particularly sensitive to this phenomenon, with a limiting rate of replication and high levels of oxygen consumption; this explains the harmful cardiovascular consequences of aging [60]. In this contest, our results suggest that the mTOR inhibition could be considered an important regulator of oxidative stress by promoting mitochondrial biogenesis and function. Finally, we evaluated two pivotal markers of inflammaging [61] in two groups of patients to verify the anti-aging effect of mTORi on these biomarkers. Our findings showed that the presence of mTORi was associated with a reduction of the above markers, indicating that blocking the mTOR pathway reduces or delays age-related biomarkers. As is well known, PTX3 is a specific marker of localized vascular inflammation and damage, because it is synthesized by cells directly involved in atherosclerosis such as macrophages smooth muscle cells and endothelial cells and [62]. PTX3 as a pivotal role in in innate immunity as a soluble pattern recognition receptor; moreover, PTX3 is localized in atherosclerotic lesions [63]. PTX3 can induce tissue factor expression in monocytes and endothelial cells [64] therefore contributing to thrombosis through this mechanism. In addition, PTX3 was associated with CVD, all cause death and CVD risk factors in a large cohort of patients [65]. Finally, our results supported the findings of Flynn et al. that demonstrated, in animal models, that rapamycin-based therapy can extends the lifespan of mammals with functional benefits to several tissues such as improvement in contractile function, antihypertrophic signaling in the aged heart and reduction of age-related inflammation [66]. In addition, p21^ink^ is known as a cyclin-dependent kinase inhibitor with a pivotal role in renal aging at the level of tubular epithelial and leucocytes; our data demonstrated that mTORi could reduce the activity of this kinase with positive effects on cellular homeostasis and vitality.

Potential study limitations include its retrospective nature and the relatively small number of patients, other than the fact that no major CV events occurred during the two years of follow-up that prevent us doing a direct conclusion. Conversely, kidney graft recipients with CKD constituted our pts’ population. These subjects share the same or even a greater, CV risk and that the use of steroids and CNI had influenced our observation; since we do not plan to withdraw these drugs, the effects observed were uniquely due to mTOR inhibition and this represent a key strength of our study. Moreover, the main problem with the use of mTORi is the high drop-out rate due to side effects. However, with dose-proposed, none patients were drop-out.

In summary, our findings represent, the first attempt, to our knowledge, to investigate the ability of chronic pharmacological mTOR inhibition to delay vascular changes in a human model of accelerated vascular aging like KTRs. In addition, our results may shed light on the mechanisms involved in the pathogenesis of CKD-induced CVD and clarify, in a clinical setting, the links between mTOR activation and other relevant regulatory systems in aging biology as klotho/FGF23 axis and mitochondrial function/biogenesis.
